# Supplementary material for: CD4 downregulation precedes Env expression and protects HIV-1-infected cells from ADCC mediated by non-neutralizing antibodies
Source: mBio. 2024 Oct 7;15(11):e01827-24. doi: 10.1128/mbio.01827-24 (PMC11559134; doi:10.1128/mbio.01827-24)
Supplement: Supplemental Figures — Figures S1 to S7. [file mbio.01827-24-s0001.pdf]

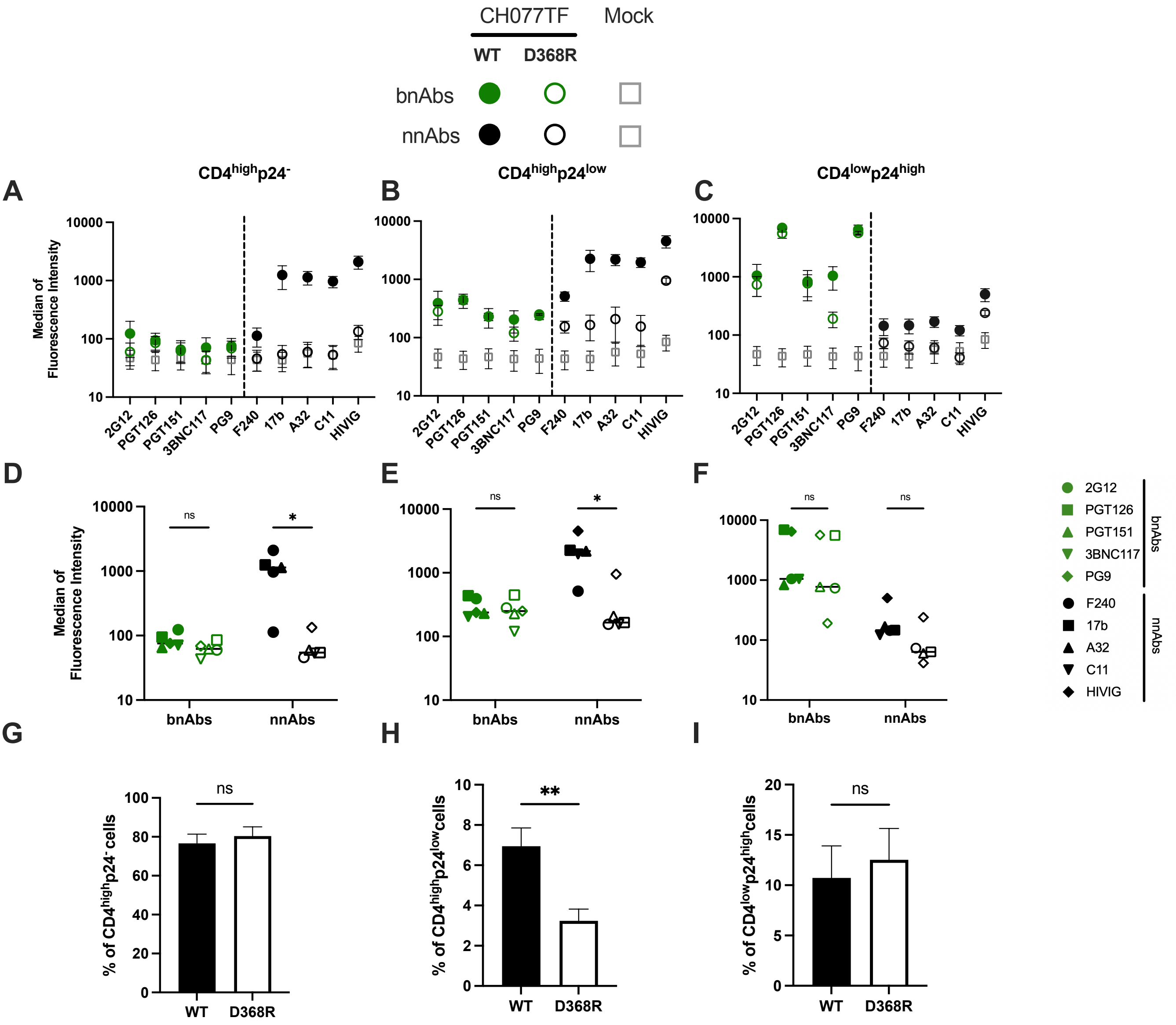


**Supplemental Fig. 1. Impact of the D368R mutation on nnAbs and bnAbs binding.**
Primary CD4+ T cells, mock-infected or infected with the transmitted-founder virus CH077, either expressing the wild-type (WT) or D368R Env (D368R) were stained with a panel of bnAbs and nnAbs, followed with appropriate secondary Abs. Cells were then stained for cell-surface CD4 prior detection of intracellular HIV-1 p24. (A) Graphs shown represent the median fluorescence intensities (MFI) obtained for at least 3 independent staining with the different mAbs. Error bars indicate means standard errors of the means. (B) Graphs shown represent the mean MFI obtained with each mAbs. (C) Percentage of the different cell populations at 48h post-infection. Statistical significance was tested using Mann-Whitney U test (** p<0.01, ns: non-significant).

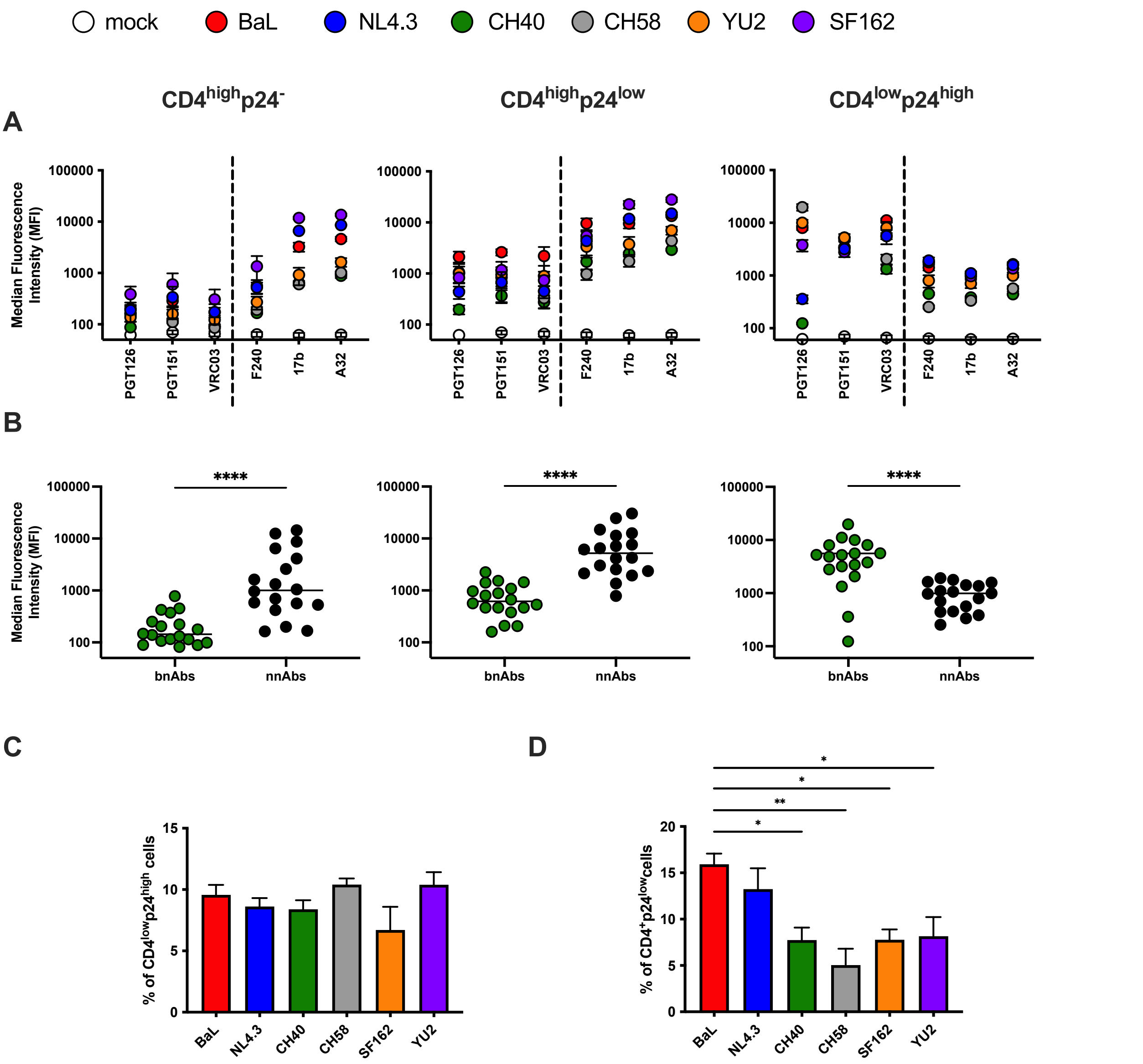


**Supplemental Fig. 2. Recognition of cells infected with HIV-1 constructs expressing lab-adapted and primary Envs by bnAbs and nnAbs.**

Primary CD4+ T cells, mock-infected or infected with NL4.3 infectious molecular clones (IMC) expressing Env from lab-adapted (BaL, NL4.3) or primary (CH040TF, CH058, YU2, SF162) viruses were stained with a panel of bnAbs and nnAbs, followed with appropriate secondary Abs. Cells were then stained for cell-surface CD4 prior detection of intracellular HIV-1 p24. (A) Graphs shown represent the median fluorescence intensities (MFI) obtained for at least 3 independent staining with the different mAbs. Error bars indicate means +/- standard errors of the means. (B) Graphs shown represent the mean MFI obtained with each mAbs for each HIV-1 IMC. (C-D) Percentage of the (C) $CD4^{low}p24^{high}$ and (D) $CD4^{high}p24^{low}$ populations at 48h post-infection. Statistical significance was tested Mann-Whitney U test (* p<0.05, ** p<0.01, **** p<0.0001).

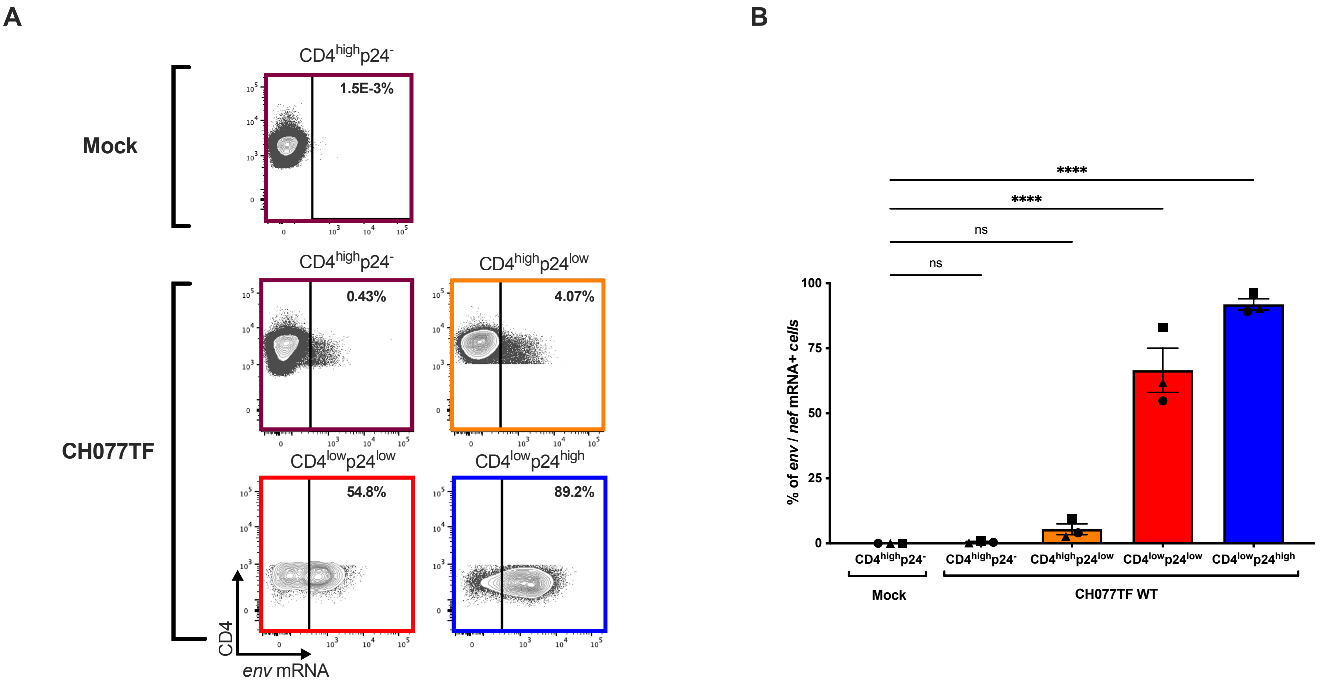


**Supplemental Fig. 3. CD4 downregulation precedes Env expression.**
Primary CD4+ T cells, mock-infected or infected with the transmitted-founder virus CH077 WT were stained for cell-surface CD4 prior detection of intracellular HIV-1 p24 and Env mRNA and nef mRNA by RNA-flow FISH. (A) Example of RNA-flow FISH detection of env mRNA among the $CD4^{high}p24^{-}$, $CD4^{high}p24^{low}$, $CD4^{low}p24^{low}$ or $CD4^{low}p24^{high}$ cell populations. (C) Quantification of the percentage of env mRNA+ cells detected among the different cell population with three different donors. Statistical significance was tested using one way ANOVA with a Holm-Sidak post-test (* $p<0.05$, ** $p<0.01$, ns: non-significant).

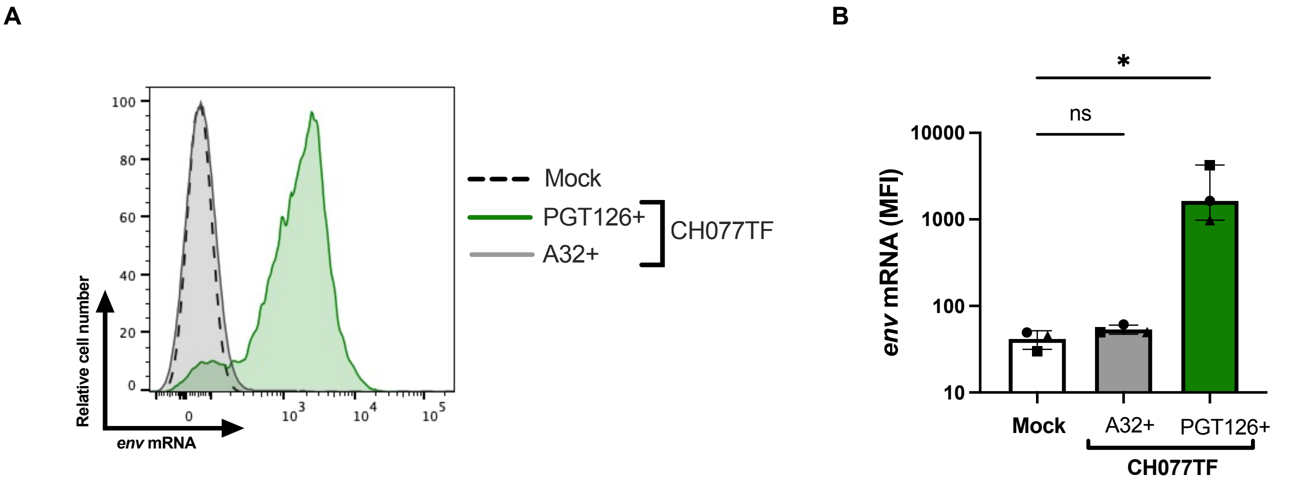


**Supplemental Fig. 4. Cells targeted by A32 are *env* mRNA negative.**

Primary CD4+ T cells, mock-infected or infected with the transmitted-founder virus CH077 WT were stained with A32 or PGT126, followed with appropriate secondary Abs prior detection of intracellular *env* mRNA by RNA-flow FISH. (A) Histograms depicting representative *env* mRNA detection on A32+ or PGT126+ cells. (B) Median fluorescence intensities (MFI) of *env* mRNA obtained with 3 different donors. Statistical significance was tested using a Kruskal-Wallis test with a Dunn's post-test. (* $p<0.05$, ns: non-significant)

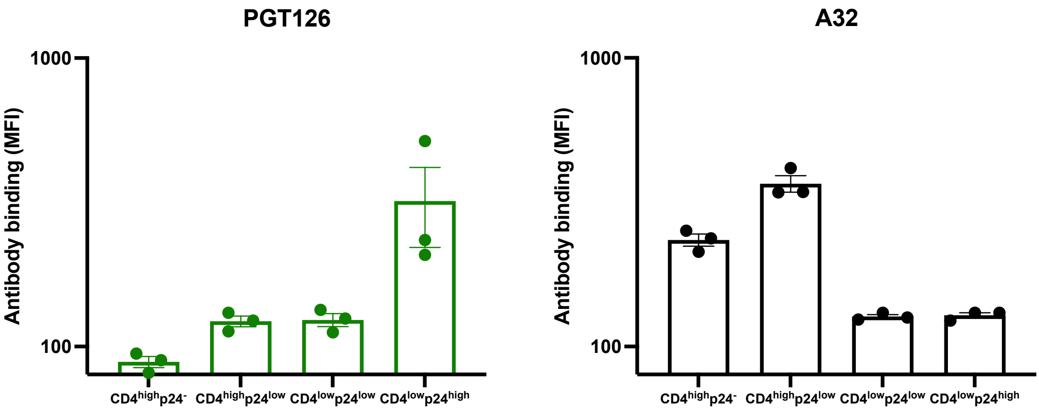


**Supplemental Fig. 5. mAbs binding based on CD4 and p24 detection.**
Primary CD4+ T cells, mock-infected or infected with the transmitted-founder virus CH077 WT were stained for cell-surface CD4, A32 and PGT126 prior detection of intracellular HIV-1 p24 and *env* mRNA and *nef* mRNA by RNA-flow FISH. Shown are the levels of A32 and PGT126 binding among the $CD4^{high}p24^{-}$, $CD4^{high}p24^{low}$, $CD4^{low}p24^{low}$ or $CD4^{low}p24^{high}$ cell populations using the same gating strategy presented in Fig. 3A.

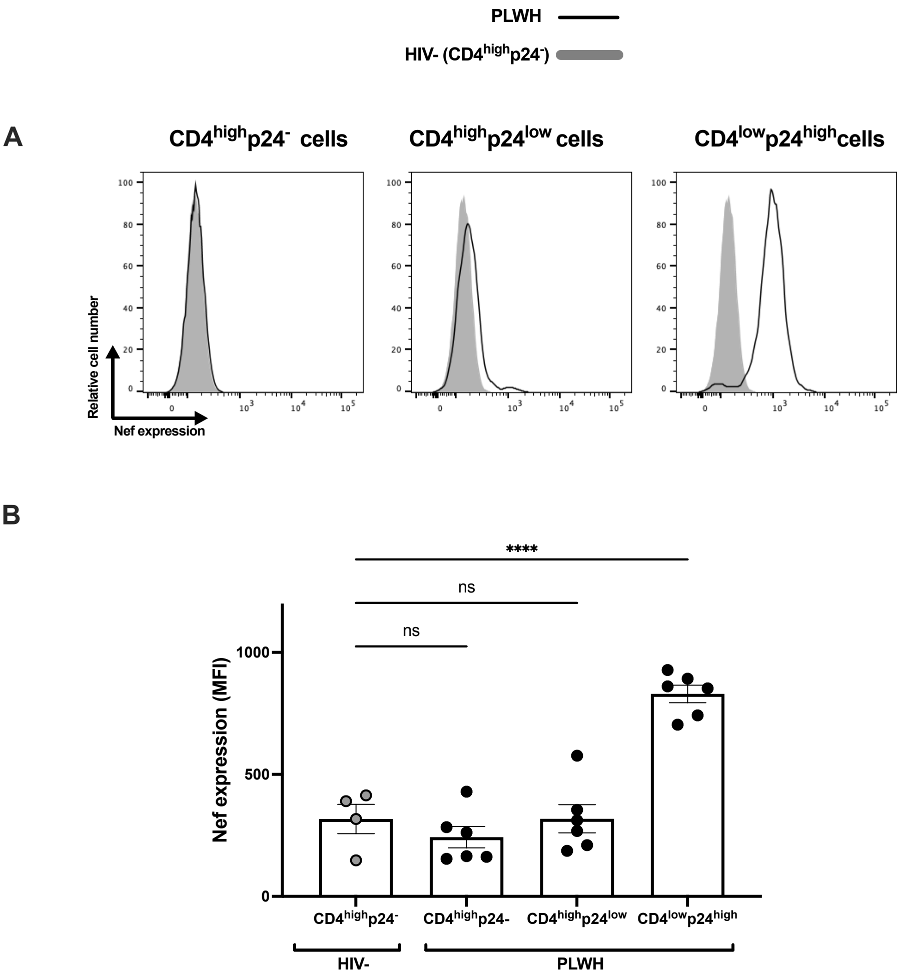


**Supplemental Fig. 6. Nef expression in ex vivo expanded CD4+ T cells isolated from PLWH.**
Ex vivo expanded CD4 T cells from 6 PLWH and 4 HIV- individuals were stained for surface CD4 prior to detection of intracellular Nef and p24. (A) Histograms depicting representative staining. (B) Median fluorescence intensities (MFI) obtained with 6 PLWH and 4 HIV- individuals. Statistical significance was tested using one-way ANOVA test with a Holm-Sidak post-test (**** p<0.0001, ns: non-significant).

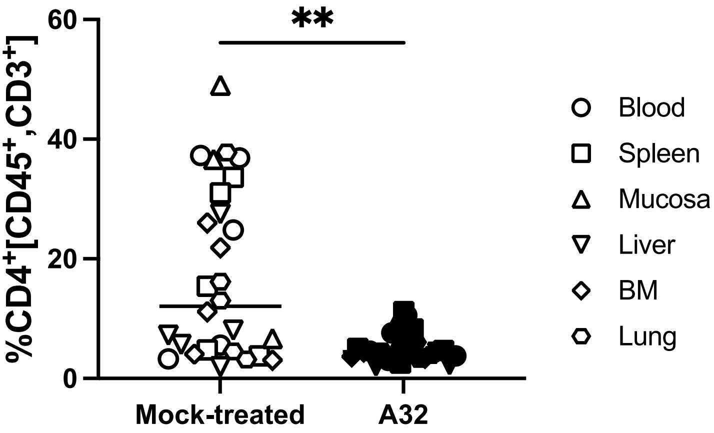


**Supplemental Fig. 7. A32 reduces the levels of CD4+ T cells in tissues in vivo.**
NSG-15-Hu-PBL mice were infected with HIV-1 JRCSF intraperitoneally. At day 6 and 9 post infection, mice were administered 1.5 mg of A32 mAb subcutaneously (s.c.). The percentage of CD4+ T cells was evaluated by flow cytometry in blood, spleen, mucosa, liver, bone marrow (BM) and lung at day 11 post-infection. Statistical significance was tested using a Mann Whitney U test (** $p<0.01$).
