# Supplementary figures and images for: CD4 downregulation precedes Env expression and protects HIV-1-infected cells from ADCC mediated by non-neutralizing antibodies
Source: mBio. 2024 Oct 7;15(11):e01827-24. doi: 10.1128/mbio.01827-24 (PMC11559134; doi:10.1128/mbio.01827-24)

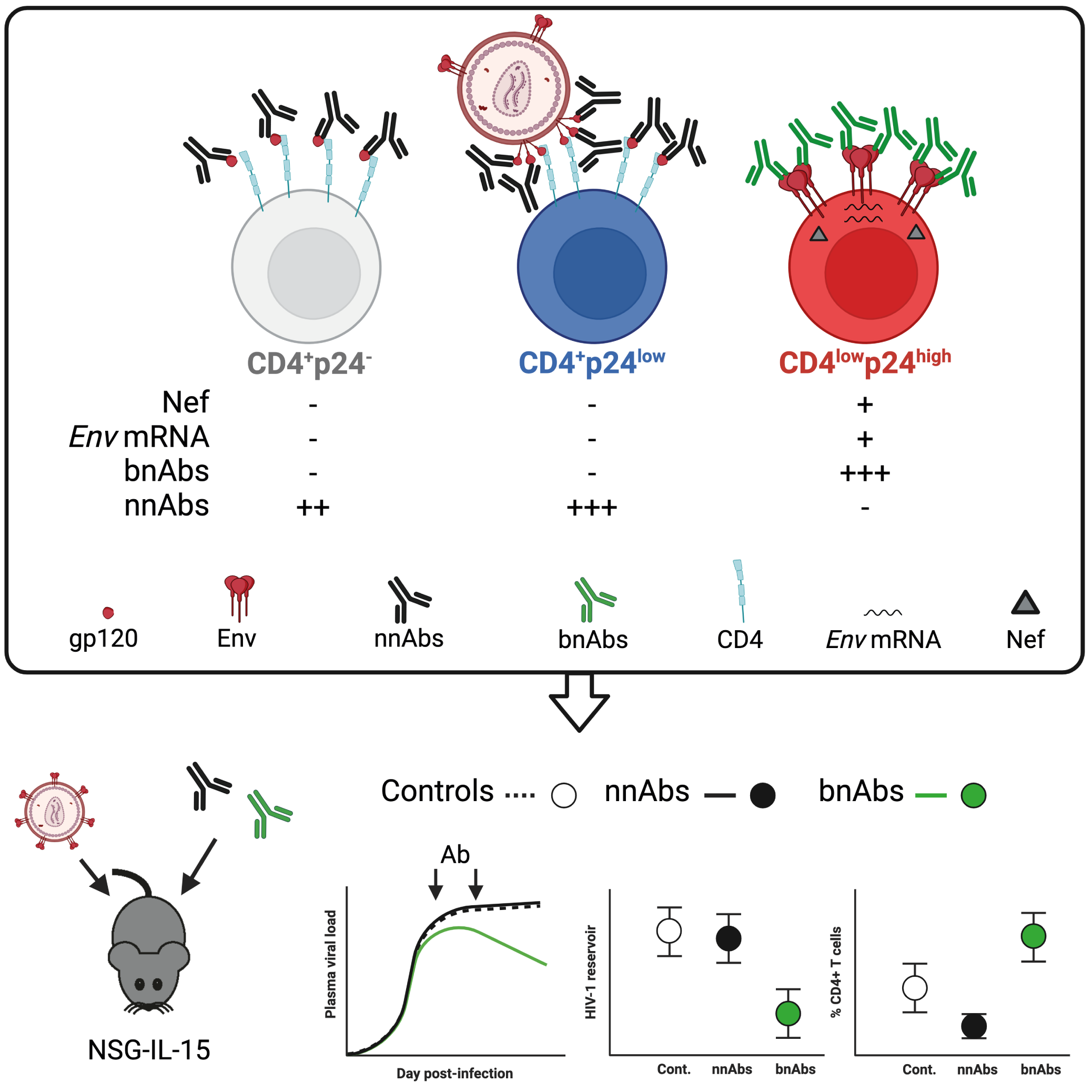

Supplement: Abstract — Graphical abstract. [file mbio.01827-24-s0002.tif]
